# Longitudinal monitoring of the mouse brain reveals heterogenous network trajectories during aging

**DOI:** 10.1038/s42003-024-05873-8

**Published:** 2024-02-20

**Authors:** Özgün Özalay, Tomas Mediavilla, Bruno Lima Giacobbo, Robin Pedersen, Daniel Marcellino, Greger Orädd, Anna Rieckmann, Fahad Sultan

**Affiliations:** 1 Department of Medical and Translational Biology, Umeå University, 90 187 Umeå, Sweden; 2 Department of Diagnostics and Intervention, Radiation Physics, Umeå University, 90 187 Umeå, Sweden; 3 Present Address: University of Groningen, University Medical Center Groningen, Department of Nuclear Medicine and Molecular Imaging, Hanzeplein 1, 9713 GZ Groningen, the Netherlands; 4 Present Address: Institute for Psychology, University of the Bundeswehr Munich, Neubiberg, Germany

**Keywords:** Cognitive ageing, Neurodegeneration, Translational research, Functional magnetic resonance imaging, Network topology

## Abstract

The human aging brain is characterized by changes in network efficiency that are currently best captured through longitudinal resting-state functional MRI (rs-fMRI). These studies however are challenging due to the long human lifespan. Here we show that the mouse animal model with a much shorter lifespan allows us to follow the functional network organization over most of the animal’s adult lifetime. We used a longitudinal study of the functional connectivity of different brain regions with rs-fMRI under anesthesia. Our analysis uncovers network modules similar to those reported in younger mice and in humans (i.e., prefrontal/default mode network (DMN), somatomotor and somatosensory networks). Statistical analysis reveals different patterns of network reorganization during aging. Female mice showed a pattern akin to human aging, with de-differentiation of the connectome, mainly due to increases in connectivity of the prefrontal/DMN cortical networks to other modules. Our male cohorts revealed heterogenous aging patterns with only one group confirming the de- differentiation, while the majority showed an increase in connectivity of the somatomotor cortex to the Nucleus accumbens. In summary, in line with human work, our analysis in mice supports the concept of de-differentiation in the aging mammalian brain and reveals additional trajectories in aging mice networks.

## Abstract (web summary)

A longitudinal aging study using fMRI in anesthetized mice suggests brain network changes like humans. Mostly, female mice show a de-differentiation of networks with increased connectivity of prefrontal to somatomotor and somatosensory networks.

## Introduction

A gradual age-related decline of most biological processes including alterations in brain structure and function have been extensively reported^1,2^. These brain changes are concomitant with cognitive impairments leading to the hypothesis that structural and functional alterations may contribute to cognitive decline in several pathological conditions, such as Alzheimer disease^3^. Most of the aging studies in human rest upon cross-sectional design which are shown to be limited in terms of estimations of age-related changes over time compared to a longitudinal setting^4^. However, this is often difficult in humans due to the long observational window required to detect age-related changes. While pathological aging is typically not found in non-humans, other mammalian biological processes display a similar gradual decline of function^5^. Given their shorter lifespan (months versus decades), rodent animal models of aging are advantageous by substantially reducing the observational window required. However, there are currently no longitudinal mice studies of changes in brain function. It is therefore not known whether the aging mouse brain shares features of human brain aging. Studying aging in another mammalian brain could allow an understanding of age-related changes that are specific for the human brain, and whether other mammals show similar vulnerability.

Advances in functional magnetic resonance imaging (fMRI) during resting-state (rs-fMRI) have revealed that several brain regions show slow, correlated, fluctuations in hemodynamic brain responses^6–8^. The application of graph theory has allowed researchers to characterize functional brain networks^9^ as a set of nodes and edges in a flexible and simple representation for whole-brain network analysis^10^. Using this approach it became possible to mathematically divide the human network into segregated groups of well-connected communities/modules, such as the default-mode network (DMN), the fronto-parietal, somatosensory-motor networks and the dorsal and ventral attention networks^7^. Network analyses can detect common features, such as hubs, number of edges connecting nodes, modules of closely related nodes, and importantly, allows analyses to relate changes in the hubs/nodes connectivity to different neuro-psychiatric diseases^11^. The combination of rs-fMRI and network analysis have revealed an important facet of human aging: changes in the degree of integration and segregation of functional brain networks^12^. In human aging, the degree of segregation of these networks becomes blurred, characterized by a reduction of within module connectivity and an increase of between module connectivity^12,13^. Changes in whole brain graph properties during aging have also been described regarding parameters as local efficiency and modularity^14,15^. Such graph properties are less dependent on module detection as required for the segregation index. While the relationship between network changes during aging and cognition is not fully understood, it has become clear that changes in module configuration are neurological hallmarks of cognitive decline ^16,17^.

Studies in rodents have reported the typical resting state networks. A consensus paper^18^ on rodent rs-fMRI showed large similarities in data acquired from various groups under different conditions (e.g., MR setup, anesthesia). A connectivity analysis of the mouse brain from 17 data sets revealed multiple components consistent with a latero-cortical network (somatomotor and somatosensory areas), the DMN (prefrontal, cingulate/retrosplenial, and temporal associative areas) and the insular area (AI). Egimendia and colleagues^19^ extended the analysis to include older animals in a cross-sectional functional connectivity study of 2 to 13 months old C57BL/6 J male mice, covering young-adult to middle age^20^. Interestingly, they found globally decreased functional connectivity in middle-aged mice (12–13 months) compared to 8–9 months mice. However, cross-sectional age-related differences might deviate from longitudinal trajectory and thus longitudinal studies are called for to characterize the trajectory of age-related changes, particularly for old mice (18–24 months). Longitudinal studies of functional connectivity in older rodents are difficult, due to the potential fragility of such animals. Given the substantive benefits of investigating aging in animal models, longitudinal evaluations are desperately needed to clarify the general biological processes underlying human aging. Thus, in this study, we aim to extend the rs-fMRI analysis to show that the mouse animal model is usable for longitudinal monitoring of brain activity in old mice. More specifically, we set out to estimate age-related changes in brain functional organization using graph theoretic approach akin to those used in human brain aging analysis of network dynamics, i.e., analysis of community/module de-segregation. Furthermore, we also wanted to look at the effect of aging on additional network properties (node and edge properties) to uncover any critical hubs and whether these nodal graph measures can uncover any brain regions that are most associated with brain aging.

## Results

### Age-related network reorganization

**Fig. 1:**
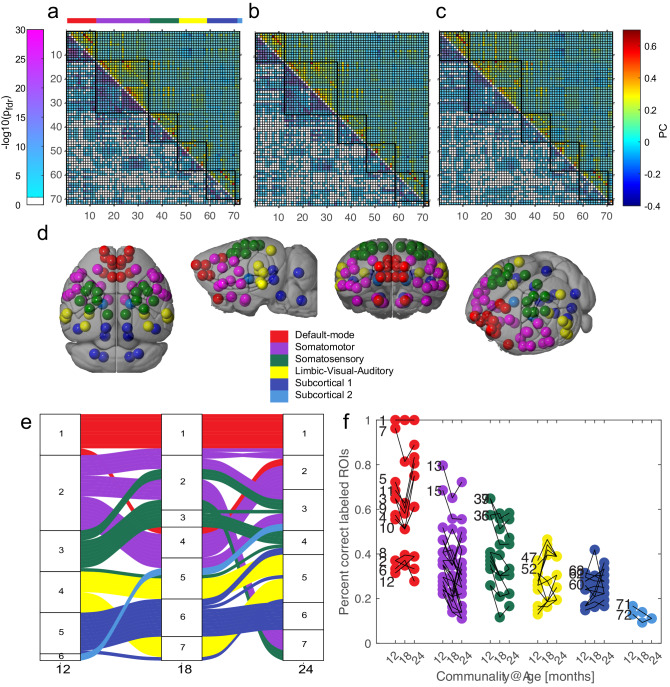
Reproducibility and dynamics in mice aging brain networks. **a**–**c** Mean ROI to ROI Fischer’s transformed z-score including both positive and negative correlations are shown in the upper triangular part for each time point. **a** 12 months (total/male/female: 50/33/17), (**b**) 18 months (47/31/16) and (**c**) 24 months (36/20/16). The lower triangular part shows the −log10 of the *p*-values derived from an independent one-sided *t*-test for each pair of ROIs (threshold *p* < 0.05, FDR corrected). The color bar on the left relates to the lower triangular part (−log10 *p*-values) and on the right to the correlation *z*-scores. **d** 12 months Louvain modules. Modules and their ROIs are color coded and plotted at their mean spatial location. **e** Trajectories of computed Louvain modules during aging. Modules display different stability. The six modules at 12 months split, diverge, and rejoin into 7 modules at 18 and 24 months. **f** Individual level reproducibility of Louvain modules: The frequency that a ROIs was correctly assigned to its module was quantified and is plotted for the three time points at which the mice were scanned. Most ROIs changed their percentage of correct assignment. However, equal numbers showing a decrease as well as an increase in correct assignment between the time points. Some of the largest increases were observed at later ages (18 and 24 months) in the first two modules. This includes the prelimbic and orbital areas of the 1st module (e.g., prefrontal/DMN) and caudoputamen and pallidum of the second somatomotor module (see Supplementary Data 2 for descriptions and ROI number assignment).

Using Louvain clustering, we defined network partitions based on individual functional connectivity matrices across three time points corresponding to 12, 18, and 24 months of age. Group-average connectivity matrices are depicted for each time point in Fig. 1a–c. The lower triangle depicts thresholded networks based on one-sample t-tests (different from zero; *p* < 0.05; FDR-corrected). We used baseline observations, i.e., 12 months, to classify a group-level consensus partition, yielding six networks (Fig. 1d). The first three networks largely corresponded to prefrontal/DMN, somatomotor, and somatosensory networks, while the latter three networks depicted more integrated structures, including a limbic-visual-auditory network, midbrain-cerebellar, and bilateral thalamus. A complete list of nodes and network associations are presented in Supplementary Data 1. To investigate the classification consistency of our network architecture, we quantified the number of iterations for which individual nodes were correctly assigned to their consensus partition. Greater consistency in network assignment reflects greater network-specific connectivity, while lower classification consistency implies that a node expresses greater integration between two or more networks. We observed the greatest consistency within DMN, somatomotor, and somatosensory networks, while the limbic and two subcortical networks expressed larger classification inconsistencies. This indicates that subcortical nodes, particularly thalamus, midbrain and cerebellum, exhibit greater network integration compared to their cortical counterparts. Next, we set out to investigate age-related reorganization in network architecture. To this end, we compared network partitions computed at 12, 18, and 24 months of age. The latter two time points yielded seven networks. Changes in network architecture and its reorganization are presented in the flow charts in Fig. 1e with colors illustrating network assignment at baseline. We observed that the DMN and midbrain-cerebellar networks remains relatively stable across all three time points, while somatomotor and somatosensory regions merge into two integrated networks at 18 and 24 months compared to baseline.

We further compared our correlation matrices at 12 months to previous rsfMRI results using the same Atlas ROIs^18^. Grandjean and colleagues used an independent component analysis and derived 13 ICA and the ROIs’ loadings on these components. We compared the functional connectivity of ROIs that had large loading on an ICA (threshold value 1.5 independent component analysis loading) with their ICA scoring (Supplementary Fig. 1). The comparison confirms that higher independent component analysis scores are associated with positive correlations, pointing to similar results from the two analyses.

Taken together, Louvain communalities can be detected at 12 months that in part are like those reported previously, i.e., DMN, somatomotor, and somatosensory networks. The flowchart depicting the communalities at 12, 18 and 24 months shows variability in the communalities during aging. Next, we want to quantify the changes between the communalities.

### Changes in network segregation during aging

**Fig. 2:**
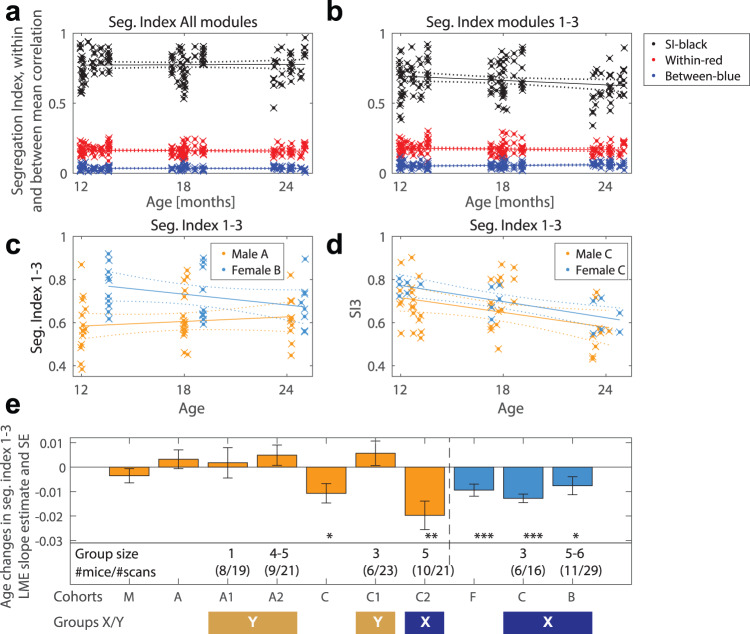
Segregation index in mice aging brain networks. The segregation index (SI) was computed at different ages by using the Louvain modules classification obtained at 12 months. This allows to compare within and between functional connectivity and the influence of age. The segregation index (**a** and **b** in black) from the within (**a** and **b** in red) and between (**a** and **b** in blue) module resting state functional connectivity’s is calculated for each individual using positive correlations only. No statistically significant changes in the SI were detected when all modules were included (**b**: Age: *t* = 0.16, *p* = 0.88, df=129). When only the first three modules were taken into consideration (SI3 in **b**) a significant decline in the SI3 (Age: *t* = −2.24, *p* < 0.027, df=129) during aging was evident, mainly due to an effect of female mice which showed a significant decrease as revealed when we looked at the interaction between age and sex (SI3~ ~ Age * Sex + (1 | ID): *t* = −1.2, *p* = 0.23; Sex (Female): *t* = 2.5, *p* < 0.013; Age/Sex: *t* = −1.5, *p* = 0.13). We next tested the effect of sex and group composition by looking at different cohorts (**c**–**e**) as defined by different vendors and group size composition. A comparison of our two cohorts that were obtained as adults from the vendors (**c**) confirmed that females showed a decline in the SI3 (Age: *t* = −2.06, *p* = 0.0488, df = 27). The female result was also confirmed in the cohort that was bred at the local animal facility (**d**) with age showing a significant effect on the decline of SI3 (age: *t* = −7.3, *p* = 0.000003, df = 15). Male cohorts largely did not show a significant decline in the SI3, except for one cohort. The male cohort C (Fig. 2d) was further divided into a cohort with a group size of 3 (C1) and one with group size of 5 (C2), with the former showing a non-significant increase in SI3 (*t* = 1.13, *p* = 0.28, df = 11) and the latter a significant decrease in SI3 (*t* = −3.39, *p* = 0.003, df = 19). Figure **e** depicts the slopes of the LME fits and shows the regrouping of cohorts A1, A2, and C1 into group Y and male C2 with females C and B into group X. Figure **e** also includes the groups sizes of the cohorts and the number of individuals (#mice) and total number of scans per cohort. Error bars in **e** are the LME standard error of the coefficient estimate.

To investigate age-related changes in within and between-modules reorganization (i.e., functional connectivity matrices depicted in Fig. 1a–c), we calculated the segregation index. Age was defined as days between scan date and date of birth. Results are presented in Fig. 2a, showing the segregation index and within and between mean ROI-ROI correlations. Including all six modules found at 12 months into the segregation index (SI) does not reveal a significant effect of age (*t* = 0.156, *p* = 0.88). However, limiting the analysis to the most reliable modules based on the percentage of correct labeling (Fig. 1f), we obtained the SI for the first three: prefrontal/DMN, somatomotor, and somatosensory networks (SI3). This now reveals a significant decline in the segregation index (Fig. 2b: *t* = −2.24, *p* = 0.026) and is based on the combining effects of a non-significant decrease in the within functional connectivity (Fig. 2b: *t* = −1.2, *p* = 0.23) and increase in the between module connectivity (Fig. 2b: *t* = 1.61, *p* = 0.11). The SI3 calculation also revealed six mice that had very low SI3 at 12 and 18 months (below 0.43) that furthermore either developed neurological symptoms or died early on of unknown reasons (listed in Supplementary Table 1 and Supplementary Data 2). These mice were therefore excluded from further analysis. We also tested the effect of multiple anesthesia’s on network segregation and found no statistical effect on the SI3 (see Supplementary Information, Supplementary Note 1).

Statistical modeling the interaction of sex and cohort on aging, revealed no significant interaction between sex and age, and in contrast a significant interaction of cohort membership and age with cohort C showing the largest interaction with age (*t* = −3.28, *p* = 0.0014). In a next step we stratified our results according to the animal’s sex and cohort affiliation (Fig. 2c–e) and found that female mice showed a highly significant decrease in SI3 (Fig. 2e: *t* = −3.8, *p* = 0.0004 for females, compared to *t* = −1.2, *p* = 0.23 for males). Further stratifying the mice into different cohorts showed that some male cohorts (C2) also reveal a significant decrease in SI3 (Fig. 2e: *t* = −3.4, *p* = 0.003). Therefore, we regrouped the mice into group Y (male cohorts A1/2 and C1) and group X (all females and male cohort C2).

### Global network analysis

**Fig. 3:**
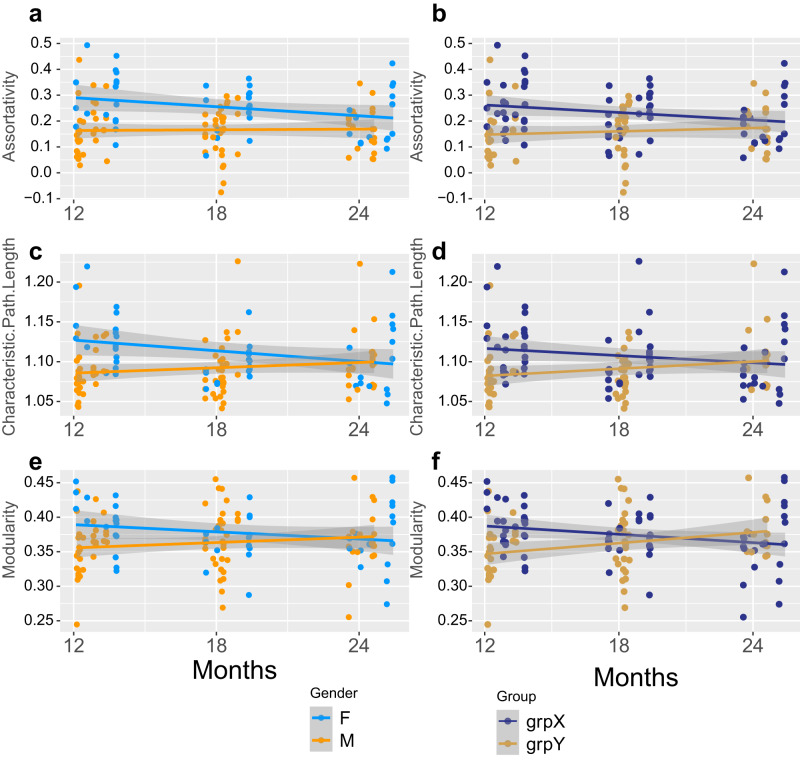
Comparison of sex and cohorts global graph networks measures. Statistical comparison using an LME model of global graph measure and age and either sex or group Y/X interaction with individuals as random factor (**a**, **c**, **e** Global graph measure ~ 1 + Age * Sex + (1 | ID) or **b**, **d**, **f** Global graph measure ~ 1 + Age * Group (Y/X) + (1|ID)). The largest effect was observed on modularity and characteristic path lengths (CPL) with significant effect of sex or group and age:sex or age:group interaction. Group X showed larger assortativity (**a**), CPL (**c**), and modularity (**e**) at an early age with a subsequent decline, while group Y showed the opposite. Data plotted are from *n* = 58 individuals and 133 observations. The detailed statistics of different graph properties are listed in Table 1. Confidence regions plotted correspond to 95% confidence intervals.

Next, we tested whether our results of network de-segregation was also supported through other global network properties. We examined six global graph metrics to assess network characteristics over time and plotted the three showing largest changes in Fig. 3. These characteristics included network integration (characteristic path length/CPL), segregation (modularity) and assortativity. We compared the different grouping: sex vs grouping X/Y (based on similar SI3 changes during aging). We did not find any significant effect of time alone on any of the graph measures (see Fig. 3 and Table 1 for details on statistics). We found significant effect of sex on CPL, modularity and assortativity. These became larger when comparing group Y vs X. We also found that CPL yielded significant interaction of age and sex in females showing a decrease in CPL during aging, while males showed a tendency to increase their CPL. The grouping X vs Y confirmed and yielded significant interactions for all three parameters with the largest changes observed in modularity, with a decrease in group X and increase in group Y. These results agree with the de-segregation as measured by the segregation index SI3 and further confirms the grouping of the mice into an X and Y groups.

**Table 1:** LME of global network properties

	Male vs. female			Group Y vs. X		
	A	M	CPL	A	M	CPL
Age (*t*)	−1.46	−0.28	−0.82	−0.75	0.39	0.01
Age (*p*)	0.15	0.78	0.41	0.46	0.7	0.99
Sex M/grp Y (*t*)	**−3.1**	**−2.3**	**−3.16**	**−3.38**	**−3.4**	**−3**
Sex M/grp Y (*p*)	**<0.0025**	**<0.023**	**< 0.002**	**<0.0011**	**<0.0009**	**<0.004**
Age*sex/grp (*t*)	1.85	1.89	**2.45**	**2.27**	**3**	**2.38**
Age*sex/grp (*p*)	0.07	0.06	**<0.016**	**<0.026**	**< 0.003**	**<0.019**

Bold *t*- and *p*-values show significant results.

### Graph network analysis – node properties

To understand age-related changes in functional connectivity beyond individual edges in the first three modules, we computed four network metrics to characterize graph properties on the nodal level. Namely, degree centrality (representing the number of neighboring nodes), betweenness centrality (hubs connecting two separate clusters), nodal efficiency (inverse of the average shortest path connecting all neighbors) and clustering coefficient. Node metrics and ROI’s that show significant changes are listed in Table. 2. The measures that yielded both similar results and were most sensitive were nodal efficiency and degree of centrality. Combining left and right areas and looking at the changes through an LME analysis showed that areas that decreased their connectivity were the secondary somatosensory (Sss) and the primary somatosensory area (Ssp.n), while the Nucleus accumbens showed an increase in nodal efficiency/degree of centrality. These changes were confirmed in group Y, while group X showed a different trend by increasing the nodal efficiency of the prelimbic cortex and anterior cingulate area.

**Table 2:** LME of nodal properties

Nodal efficiency (All)			Degree centrality (All)			Betweenness centrality (Grp X)		
Ssp.n	−4.4	0.0011	Ssp.n	−3.67	0.0087	PAL	−3.6	0.014
Sss	−6.1	0.0000	Sss	−5.7	0.0000	**Clustering coef (All)**		
ACB	4.6	0.0005	ACB	3.62	0.009	ACB	4.4	0.0006

Nodal efficiency (Grp Y)			Degree centrality (Grp Y)			Clustering coef (Grp Y)		
Sss	−4	0.0053	Sss	−4.7	0.0007	ACB	4.1	0.0001
ACB	3.54	0.023	ACB	4.4	0.0013	–	–	–

Nodal efficiency (Grp X)			Degree centrality (Grp X)			–	–	–
PL	3.9	0.006	PL	3.4	0.027	–	–	–
ACA	3.49	0.02	ACA	3.2	0.045	–	–	–

ROI abbreviations are listed in Supplementary Data 1; *t*-stat, *p*-value (FDR corrected).

**Fig. 4:**
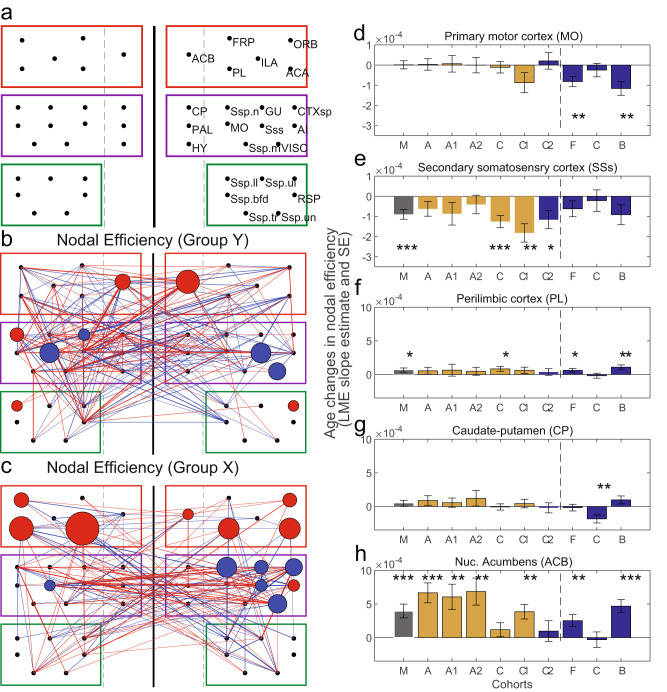
Comparison of functional connectivity with nodal graph measures. **a**: First three Louvain modules obtained at 12 months are shown grouped by color coded rectangles (left and right hemisphere sides separated by thick line). Stippled lines separate cortical versus subcortical brain regions. In panel **b** (group Y) and **c** (group X) we plotted the nodal efficiency modulation with age as discs of varying diameter and the ROI-ROI connectivity’s that also showed modulation with age (thresholded at *p* < 0.01, uncorrected). Increases of nodal efficiency or connectivity strength are plotted in red, while decreases are shown in blue with size and thickness corresponding to the modulation strength. Evident is the decrease in somatomotor nodal efficiency (**b**, **c**) together with major decreases in interhemispheric connectivity, while there are some increases in prefrontal/DMN connectivity to the somatomotor modules. In group Y these mainly target the N. accumbens which also shows increases in its nodal efficiency. Group X show an increase in the nodal efficiency of the prefrontal/DMN nodes (strongest in prelimbic cortex) accompanied by major increases in the connection to most other modules. ROI abbreviations are listed in Supplementary Data 2. In **d**–**h** we plotted five different nodes and their age-related changes in efficiency. This allowed us to compare node efficiency in different cohorts. We took the mean of the left and right side and plotted the nodes that showed the most conspicuous changes in **b** and **c** (**e**: supplemental somatosensory area; **f**: prelimbic area; **h**: N. accumbens), and for comparison two regions that showed fewer changes (**d**: somatomotor areas and **g**: caudate-putamen). The plots confirm that somatomotor area and caudate-putamen show the fewest significant changes, while the N. accumbens shows most changes within group Y. Furthermore, group Y showed significant decreases in the nodal efficiency of the supplemental somatosensory area (**e**), mainly due to changes in both C cohorts. In contrast, group X showed an increase in the nodal efficiency of the prelimbic area, mainly due to larger changes within the female B cohort (**f**). Number of individuals and observations are the same as in Fig. 2e. Error bars in d–h are the LME standard error of the coefficient estimate.

In Fig. 4a–c we added LME edge statistics (same as plotted in Supplementary Figs. 2) to the results of nodal efficiency. Increases of nodal efficiency are plotted in red, while decreases are shown in blue with size and thickness corresponding to the modulation strength (thresholded at *p* < 0.05, FDR corrected). Evident is the decrease in supplemental somatosensory areas’ nodal efficiency in group Y (Fig. 4b) and an increase in the connectivity of the Nucleus accumbens. The changes in edge statistics were quantified in Supplementary Fig. 2d–n. The strongest effects were found in slope increases in within-module connectivity in group Y (ks stat 0.48, *p* = 0.0001). While group X showed the strongest effects in slope decreases (ks2stat 0.41, *p* = 0.0001) in within module connectivity (Supplementary Fig. 2k), mainly due to a decrease (ks2stat 0.41, *p* = 0.0001) in intrahemispheric connectivity (Supplementary Fig. 2m). The largest number of changes were observed in group Y in the between module connectivity (Supplementary Fig. 2l: 660 vs. 300 counts) and these were mainly due to decreases (Supplementary Fig. 2n) in interhemispheric connections (474 counts in Y vs. 204 in X).

Group X showed an increase in the nodal efficiency of the prelimibic prefrontal/DMN node (Fig. 4c, f) accompanied by increases in the connection to most other modules (Fig. 4c). Figure 4 also compares the nodal efficiencies within the different cohorts. Evident is that the secondary somatosensory cortex and Nucleus accumbens show the largest changes and that they are present in most male cohorts (Fig. 4e, h). In contrast, females display stronger changes within the prelimbic cortex, but these are restricted to the B cohort.

## Discussion

In vivo longitudinal analyses of brain networks are critical to understand brain aging^1,4,21^. In humans, longitudinal studies are difficult; nevertheless, few if any longitudinal fMRI of aging mammalian brains have been conducted so far. Here we show that adult lifespan imaging approaches in mice are feasible and that they can provide additional insights into altered brain network organization at an advanced age.

One challenge in animal longitudinal studies is drop-outs due to experimental-related intervention (e.g., anesthesia or surgery) at old age. In our experience, using light anesthesia with α2-adrenoreceptor agonist medetomidine and low isoflurane^22^ could limit this loss to about 12% up until old-age and allows for repeated anesthesia (up to 10) and long data acquisition durations (up to two hours). A limitation of our approach is the alteration of network activity due to anesthesia. Recent studies, however, have shown that light anesthesia such as used in our study retains similar network activity in cortical DMN and frontal networks as in the awake in rodents^23,24^ and in humans^25^. Critically, these networks are important in studying brain aging^26^. A different result was obtained by Paasonen and colleagues^27^ using other anesthesia. They showed that rats urethan or propofol anesthesia had larger similarity to their awake rat networks compared to medetomidine-low isoflurane. This contrasts with a meta-analysis that showed that in mice the medetomidine-low isoflurane combination showed similar high functional connectivity’s as in the awake condition^18^. The recent study by Gutierrez-Barragan and colleagues has successfully used rs-fMRI in awake mice at a younger age (<6 months) by head-post implantation and habituating the animals to the MR environment. This may then allow for an additional path to monitor further aspects of aging brain networks.

Another critical aspect of using rodent animals for studying aging brain is the translational potential of the results to relevant findings in humans. We therefore selected our data analysis approach to be in line with those used in prevalent human aging studies: Atlas-based functional connectivity of ROIs and subsequent differentiating into modules to distinguish within and between-module connectivity^12^. Our Louvain module analysis revealed stable components in the first three modules, allowing to study within and between module connectivity.

A major finding in our study is that we have two patterns of brain network changes during aging. In female C57BL/6J mice there is an increase in between-module connectivity and an opposing trend of connectivity decline within modules. This leads to a de-segregation of brain modules and decreased characteristic path length and modularity. Most male C57BL/6 J mice show little global network changes, but a marked decline in functional connectivity of the second somatosensory cortex together with an increased connectivity of the N. accumbens. We also calculated additional global graph measures to test whether Louvain module assignment influenced the segregation index. The changes we observe in the characteristic path length, modularity and assortative are in line and confirm our de-segregation/segregation results and show that they are not biased by the module assignment.

The de-segregation of modules during aging (associated with a decline in the segregation index) is a hallmark of human brain aging^12,17^. Our study confirms that this can also be observed in mice; however, mostly in females showing a de-segregation of networks, while most males show few changes in network segregation. Nevertheless, in all our analysis that we refined to animals grouped by their different cohorts, the sex effect was non-stringent with for instance some males also showing a de-segregation of their modules during aging. Therefore, additional factors such as the social environment may also shape the networks trajectory during aging. We grouped animals into different cohorts based on vendor source, cage affiliation and group size as a means to verify the sex effects. This was done under the assumption that mice from same source that grow old together in the same cage will share a similar upbringing and social environmental influences. Different social environments could be for instance shaped by different levels of aggression that would also be expected to be more prevalent tin male C57BL/6 J mice^28^. Furthermore, recent studies confirm the emergence of individuality within inbred female C57BL/6 N mice and point to additional sources of variation and require methods quantifying social interactions covering the animals life^29^.

Human studies of brain connectivity have confirmed some sex differences in the DMN network^30–32^. Interestingly, these changes showed different trajectories with aging, with females showing either slower decline of DMN connectivity^32^ or different decline topography^31^, with anterior DMN showing a decline while posterior DMN an increase in connectivity in females. Most human studies are limited to a cross-sectional design due to the longer human lifespan. This has several disadvantages^2^ and leads to a bias to exclude individuals that will develop cognitive symptoms early on^31^. Interestingly, human studies utilizing a longitudinal design^17,33,34^ show a rather limited effect of sex on human brain aging.

A current deficit of mouse animal models is the scarcity of behavioral tests that probe the prefrontal/DMN networks functions. Clearly, primate behavior allows much closer links to human cognitive functions but retains the disadvantage of a much longer lifespan. Nevertheless, other behavioral studies of aging rodents allow us to draw some parallels to the observed male/female network reorganizations. Male Fischer 344 rats showed a larger impairment in their spatial memory during aging^35^ associated with a stronger decline in their physical activity. Higher physical activity in rodents is generally maintained over a longer period in females, with males showing a stronger decline^36,37^. In mammals, females generally live longer than males due to various factors including a full duplicate of chromosomes, protective hormones^38^, less aggressive social interactions^39^ and more social communications^40^. It is conceivable that at least some of these factors (e.g., enhanced demands of social interactions) show a similar influence on network aging leading to a similar aging trajectory as in humans. Future studies are required (measuring hormone levels, studying cellular effects of hormones in aged mice brains, evaluating social interactions during aging) to understand the effect of some of these factors in females, while other studies are needed to differentiate the factors (e.g., hormones, vascular and social aggression/interaction) that are important in male brain network changes. The difference we observe in different cohorts (e.g., male C1 and C2) with some also showing a “female”/human-like de-segregation of networks during aging may point to the importance of the social environment in shaping aging in mice.

The combination of nodal and edge connectivity analysis allows additional insights on potentially important nodes and the accompanying changes in connectivity during aging. Differences due to sex and cohorts provide further links to the changes in phenotype in older mice. In most males, the N. accumbens shows an increase in its nodal efficiency bilaterally, while the secondary somatosensory cortex shows a reduction in its nodal efficiency bilaterally. This is accompanied by an increase in the functional connectivity of the somatomotor module to the N. accumbens. The anatomical connections would strongly imply that these are due to an increase in the strength of the afferent connections from cortical areas to the N. accumbens. The N. accumbens receives inputs from prefronatal, motor, somatosensory and cingulate cortices^41,42^. Many of these afferents are described as bilateral projections. In contrast, the efferents of the N. accumbens target mesencephalic and striatal structures and only indirectly reach cortical areas. A hypothesis could be, that the loss in connectivity strength of the secondary somatosensory cortex within the motor module reflects a reweighting of the connections from the other parts of the 1st and 2nd modules to the N. accumbens, with the later receiving strengthened afferents during aging. Tentatively, this may be interpreted as rearranging the 1st two modules from somatosensory driven motor control to reward driven motor behavior. Such a change could be well expected from increased food-intake in males leading to their larger weights^43^.

In contrast most female mice and a subset of males display a different path. Here we either have a mainly global network de-differentiation (as seen in female cohort C) or the additional involvement of the prelimbic cortex with an increase in it’s nodal efficiency (female cohort B).The ROI-ROI edge analysis shows that both changes are due to an increase in the connectivity to the other modules (second and third module). Such changes are quite similar to the de-differentiation found in human networks during normal aging^12^. However, in female mice it is not clear whether this is a sign of cognitive decline. The prelimbic cortex in mice has been shown to be involved in cognitive functions such as spatial^44^ and working memory^45^ and delayed response tasks^46^, tasks similar to that of the primate prefrontal cortex^47^. It is therefore conceivable that the engagement of such an area in assisting other brain functions (e.g., somatomotor) may put an additional burden on its original functions. Further studies will be required to assess the cognitive burden of such an engagement.

## Methods

### C57Bl/6J cohorts

We used 65 C57BL/6 J mice in this study from three cohorts. The first cohort (Cohort A) of 22 male mice were purchased at six months age from Jackson Laboratory. The second cohort (Cohort B) comprised of 11 females was purchased from Charles-Rivers (UK) at five months age. The third cohort (Cohort C), 20 males and eight females, was bred at the Umeå Centre for Comparative Biology (UCCB) and originated from four females and one male also purchased from Charles-Rivers (UK). An additional four mice were supplemented to the 24 months group to increase the number of females (also from Charles-Rivers UK). Supplementary Table 1 and Data 2 lists the mice cohorts, source, and numbers.

During the study animals were regularly screened for any pathological signs and their body weights were regularly controlled. Mice showed a gain in weight in an expected sex-dependent fashion (see Supplementary Fig. 3). During the observational period twenty-nine mice died or were sacrificed due to health issues before reaching an age of 24-months. Less than half of those (*n* = 13) died during anesthesia. The other 16 died due to different causes such as: tumors (*n* = 2); stroke (*n* = 1); weight loss (*n* = 2); wounds (*n* = 6); not determined (*n* = 5). Prolonged high isoflurane anesthesia (longer than an hour) during structural MRI or PET/CT scans was majorly the cause of anesthesia related deaths. A change of the experimental protocol to use only low isoflurane and medetomidine reduced the number of deaths considerably: three out of 24 24-months mice died under low isoflurane and medetomidine compared to ten mice that died under the isoflurane only anesthesia (during non rs-fMRI scans).

The three cohorts underwent functional and structural MRI in three batches. Males of the first (*n* = 9) and third (*n* = 8) cohorts also underwent PET/CT experiments^48^. A summary of all animals and their functional MRI sessions (12-24 motnhs) are listed in Supplementary Data 2. To further study any effects of group composition (due to different suppliers and groups size) cohort A was further subdivided into cohort A1 (8 males that required to be housed in isolation due to aggressive behavior), cohort A2 (9 males raised in 2 groups), C1 (6 males raised in 2 groups) and cohort C2 (10 males raised in 2 groups). Summary of cohort numbers at different time points is listed in Supplementary Table 1. All mice were maintained at 21 °C temperature, 12/12 h of dark/light cycles and received water/food *ad libitum*. Food was provided as chow (1319 extrudate, Altromin Spezialfutter GmbH, Lage, Germany) in open-top cages (1284L Eurostandard Type II: L 365 × 207 × 140 mm floor area: 530 cm^2^). All procedures performed in this study were approved by the regional Animal Research Ethics Committee of Northern Norrland and by the Swedish Board of Agriculture (Ethical permit number: A17-2019).

### Anesthesia protocol and MRI acquisition

Mice were scanned at 12, 18 and 24 months (exact dates were derived from session date and date of birth and are listed in Supplementary Data 2). Functional MRI was performed solely following a dedicated anesthesia protocol^22^. After isoflurane induction (ventilation with 100% Oxygen) mice were placed onto a cryocoil-specific MRI mouse bed (Bruker, Germany) using both tooth- and ear-bars to prevent head movement during MR scans. A subcutaneous bolus injection of medetomidine (Domitor®, Orion Pharma AB, Sweden. 0.05 mg/kg) was administered to the animals and the isoflurane concentration was steadily reduced over the next two minutes from 2% to 0.5%. In addition, two minutes after the bolus injection ventilation was changed to 21% oxygen air. A constant infusion of medetomidine was provided at 0.1 mg/kg/h (s.c.), starting 15 min after the initial bolus injection to maintain anesthesia. Body temperature and respiration were monitored with SA Instruments (Model 1035, SA Instruments, Inc., Stony Brook, NY). Body temperature was measured via rectal probe and maintained at 36.5–37.5 °C) with the aid of a heating blanket and a water heating system (Pump fluid heating System, SA Instruments).

The mice were then positioned inside the MR scanner (Bruker BioSpec 94/20, Germany) with the brain in the center of the field-of-view of a cryogenic RF coil (MRI CryoProbe, Bruker, Germany). An actively shielded gradient coil (Bruker, B-GA12S HP) of 11.4 cm inner diameter was used with 220mT/m (70 μs rise time).

The animals were first scanned using a T1 FLASH sequence (TR/TE: 50/8 milliseconds; flip angle: 20°; pixel dimension: 0.125 mm isotropic). For the rs-fMRI we selected 38 coronal slices of 0.4 mm thickness to cover the brain. These slices were acquired at a temporal resolution of 1.5 s per volume with single-shot GE-recalled EPI images (TR/TE = 1500/22 ms, bandwidth=208.33 kHz, FA = 60°, FOV = 16.2 mm×9.6 mm, Matrix=54×32) and a total of 420 volumes. For additional structural MRI data acquisitions, the mice were subsequently anesthetized either using isoflurane (1.5–2% maintenance) within the same or at a different session, or with continued medetomidine anesthesia in the same session. Excess isoflurane in the MR scanner was constantly removed by a suction pump. After the scan, the mice were administered 0.3 mL of 1:20 atipamezole (Atipam, Dechra Veterinary Products, Sweden) diluted in 0.9% saline to induce awakening and rehydration. The mice were then placed into a recovery cage until fully awake, and then returned to their home cage.

### MRI preprocessing and data analysis

T1 structural images were corrected for field inhomogeneity using bias field (N4BiasFieldCorrection, ANTs v2.1) correction^49^. Individual structural/T1 brain mask were generated for each subject and session using a template-based brain extraction tool^50^. Resulting brain masks were then co-registered (FLIRT, FSL) to individual functional scan and used for skull-stripping functional data. Rs-fMRI data were preprocessed using FSL v6.0^51^ and AFNI v21.2.04^52^ software libraries. The first five volumes in each scan were removed to allow the signal to reach equilibrium. Slice timing correction (slicetimer, FSL), and motion correction (MCFLIRT^53^) was then applied. We checked on motion outlier volumes (based on relative frame-wise displacement estimations with fslmotionoutliers, FSL) and did not detect any (75th percentile + 1.5* interquartile range). Six scans were excluded in full due to the presence of artifacts or acquisition errors (see Supplementary Table 1 and Supplementary Data 2). 128 sessions were obtained with two scans (14 had one scan only due the presence of artifacts or acquisition errors in the other).

The functional data was linearly registered to the Allen Reference Atlas (Mouse Brain Common Coordinate Framework v3.0 available from atlas.brain-map.org)^54^ and was performed with FLIRT (FSL). Six estimated motion correction parameters, white matter, ventricle signals as well as mean global signal and its linear and quadratic derivatives were regressed from time series (3dDetrend, AFNI) to reduce the effects of physiological noise and motion. A high-pass temporal filter (>0.01 Hz) was applied to the time series rather than a band-pass filter, since it is shown that valuable signal might be present in higher frequencies^55,56^. Finally, images were spatially smoothed with a 0.6-mm full-width at half maximum isotropic Gaussian kernel (fslmaths, FSL).

#### Atlas-based functional connectivity

72 ROIs from Allen Mouse Brain Atlas were selected based on Grandjean et al. ^18^. This atlas is described in the study by Lein and colleagues^57^ and is based on a modified version of the Swanson^58^ and Hof atlases^59^. The atlas rois and the description files are available under Github repository, https://github.com/grandjeanlab/mergeallen. The ROIs were used to create connectivity matrices for each session by extracting average time series of the BOLD signals of all voxels in each ROI and calculating the Pearson’s correlation coefficient (r) between ROIs. Connectivity matrices were averaged in those subjects that had more than one session. Fisher’s r-to-z score transformation was applied resulting in a 72 × 72 connectivity matrix (**Z**) for each subject at each time point.

#### Louvain community/module detection

To define a representative set of functional networks, or communities/modules, we used the Louvain module detection algorithm with scripts written in MATLAB (R2021b, Natick, Massachusetts: The MathWorks Inc.) using Rubinov’s BCT package^60^ in conjunction with consensus clustering^61^. First, an initial network partition was generated for each subject, based on functional connectivity matrices using positive edges only (**Z**+). Due the Louvain algorithm’s susceptibility to local maxima, it was repeated 1000 times using an iterative modularity fine-tuning algorithm, which maximizes modularity by reassigning node-network affiliations^62^. Next, subject-wise agreement matrices were computed, representing the fraction of repetitions in which nodes were assigned to the same network. Each subjects’ agreement matrix was subsequently partitioned again until the algorithm converged to a single, subject-specific consensus partition. To define a representative group partition, an agreement matrix was computed for the subject-specific partitions, and the consensus clustering procedure was repeated until convergence of a group-level partition was reached. The procedure described above was applied for multiple resolutions, defined by the resolution parameter γ, with higher values allowing detection of smaller modules. To avoid arbitrary selection, group-level consensus partitions were computed for γ-values between 1.0–2.0, in increments of 0.1. The most representative partition was then defined as the partition with the greatest normalized mutual information between solutions^63^.

The obtained Louvain modules allowed us to calculate a network segregation index (SI) by comparing within and between connectivity^64^: ((mean within correlations – mean between correlations)/mean within correlations). This analysis was performed in MATLAB.

#### Graph network analysis

Transforming connectivity matrices (**Z**+) to binary adjacency matrices involves thresholding z-scores to retain highly correlated connections and remove spurious connections. We used sparsity or density thresholding, which keeps the same number of edges for each graph by applying a subject-specific connectivity strength threshold and therefore allowing an examination of relative network organization^65^. We used 16 different densities thresholds starting from the lowest threshold with fully connected components (0.1) to highest threshold (0.25), with steps of 0.02 for sparsity thresholding^66,67^. The following global graph parameters were calculated: clustering coefficient, characteristic path length^68^, local efficiency^69^, small world index^70^, modularity and assortativity^71^ as global metrics to assess the segregation, integration, small-world and vulnerability properties of the graphs^72^, respectively. For each measure we obtained the average over the 16 different thresholds.

Several nodal metrics including degree of centrality, nodal efficiency, clustering coefficient^68^ and betweenness centrality^73^ were analyzed for each node in the graph. The functional brain graphs were constructed using graph theory network analysis toolbox^74^. Graph metric definitions are listed in (see Supplementary Table 2 and 3).

### Statistics and reproducibility

Functional ROI-to-ROI correlations (non thresholded connectivity matrix **Z**) were averaged for each time point (12, 18 and 24 months) and were tested for significant (being different from zero) with the one-sample t-test. To investigate longitudinal changes in functional connectivity, ROI-to-ROI correlations at the three time points were analyzed using linear mixed effects (LME) model using lme4 v1.1-27.1 package^75,76^ for R v4.1.2^77^, with sex and age as fixed effect, and subject as random effect and using maximum likelihood estimations. LME can be used to analyze correlated data and therefor allows to model longitudinal data^78^. Its versatility to account for missing data points increases its statistical efficiency and makes it particularly useful to analyze longitudinal aging data^79^ where dropouts are a major concern.

We used the exact age (days to scan date from date of birth). We also included and tested sex and age interactions of nodal graph measures for each node of the atlas with LME. To estimate potential differences in Sex, we applied a contrast (see Supplementary Table 4). Time effect alone was obtained by using equal contrast for both sexes.

We used a two-sample Kolmogorov-Smirnov test (ks2test) to compare distribution and detect significant differences (*p* < 0.05). A significance value of α=0.05 was used after false discovery rate (FDR) adjustment in cases of multiple comparisons^80^. A significance value of *p* < 0.01 was also used with no multiple comparison corrections in cases where we wanted to delineate sub-threshold relationships.

### Reporting summary

Further information on research design is available in the Nature Portfolio Reporting Summary linked to this article.

### Supplementary information

Supplementary Information

Description of Additional Supplementary Files

Supplementary Data 1

Supplementary Data 2

Reporting Summary

## Data Availability

Numerical source data for all graphs in the paper have been uploaded to Dryad^81^. Exemplary functional and structural MRI raw data are available via the OSF^82^ data sharing service or upon reasonable request.

## Code availability

The scripts employed for rs-fMRI data analysis are available at Github^83^. Codes used to generate figures and statistical analysis are available upon reasonable request.
