# Supplementary material for: Longitudinal monitoring of the mouse brain reveals heterogenous network trajectories during aging
Source: Commun Biol. 2024 Feb 20;7:210. doi: 10.1038/s42003-024-05873-8 (PMC10879497; doi:10.1038/s42003-024-05873-8)
Supplement: Supplementary file 1 — Supplementary Information [file 42003_2024_5873_MOESM1_ESM.pdf]

Supplementary Table 1: Summary of mice cohorts and counts at different time points

C57Bl6J mice source and cohort numbers per age group with exclusion (quality control or physiological grounds)

**Cohort (sex)**	**Source**		**12 months**	**18 months**	**24 months**
**A1 (M)**	Jackson Laboratory (stock n°: 000664, California, U.S.A.)	Included	**7**	**8**	**4**
		QC exclude	1	0	1
		Physiol. exclude	2	0	0
**A2 (M)**	Jackson Laboratory (stock n°: 000664, California, U.S.A.)	Included	**8**	**7**	**6**
		QC exclude	0	1	0
		Physiol. exclude	2	1	0
**B (F)**	Charles-Rivers (stock n°: 000664, Kent, UK)	Included	**11**	**10**	**8**
		QC exclude	0	1	0
		Physiol. exclude	0	0	0
**C (F)**	Bred at UCCB (Charles-Rivers; stock n°: 000664, Kent, UK)	Included	**6**	**5**	**5**
		QC exclude	1	0	0
		Physiol. exclude	1	1	0
**C1 (M)**	Bred at UCCB (Charles-Rivers; stock n°: 000664, Kent, UK)	Included	**10**	**8**	**5**
		QC exclude	0	0	0
		Physiol. exclude	0	0	0
**C2 (M)**	Bred at UCCB (Charles-Rivers; stock n°: 000664, Kent, UK)	Included	**8**	**8**	**5**
		QC exclude	1	0	0
		Physiol. exclude	0	0	0

UCCB: Umeå Centre for Comparative Biology; Unclassified into either cohort were an additional 6 mice.

Supplementary Figure 1: Comparison to previous rsfMRI results

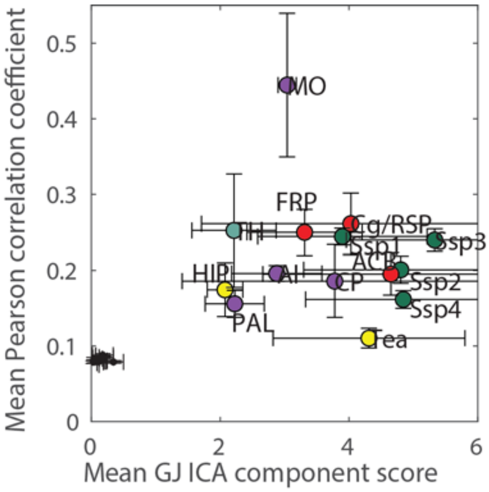

Comparison to previous rsfMRI results using the same Atlas ROIs (Grandjean et al 2020). The consensus paper derived 13 ICA and the loading of the 72 ROIs on these components. The ICA's highest loading ROIs (threshold value 1.5 ICA loading) were compared with the Fischer's transformed z-score of our aged mice and the average for each ROI is plotted. Data are color coded according to their 12 months communities/modules. We also plotted the means of the ROIS scoring less than the threshold (data in the lower left of plot). The comparison shows that the confidence intervals of ICA's score do not overlap. Error bars are the 95% confidence interval.

Supplementary Note 1

Influence of multiple anaesthesia on global graph and segregation measures

The effect of multiple anaesthesia on the results were tested by statistically modelling the number of all anaesthesia the animal had as fixed effect on either the segregation index or the global graph measure. We found no effect on either network graph outcome: characteristic path length t=1.0995, p=0.27 and segregation index (modules 1-3) t=-0.2, p=0.84. Therefore, we can exclude that the cohort which had additional PET experiments (or more MRI scans) caused the discrepancies in males.

Supplementary Table 2: Global graph metrics definition

Metric	*Formula*	*Description*
Assortativity	$r = \frac{l^{-1}\sum_{(i,j)\in L} k_i k_j - \left[l^{-1}\sum_{(i,j)\in L}\frac{1}{2}(k_i + k_j)\right]^2}{l^{-1}\sum_{(i,j)\in L}\frac{1}{2}(k_i^2 + k_j^2) - \left[l^{-1}\sum_{(i,j)\in L}\frac{1}{2}(k_i + k_j)\right]^2}$	The assortativity coefficient is a correlation coefficient between the degrees/strengths of all nodes on two opposite ends of an edge. A positive assortativity coefficient indicates that nodes tend to link to other nodes with similar degree/strength.
Clustering Coefficient	$C = \frac{1}{n}\sum_{i\in N} C_i$	Average of the clustering coefficient of all nodes.
Characteristic Path Length	$L = \frac{1}{n}\sum_{i\in N} L_i = \frac{1}{n}\sum_{i\in N}\frac{\sum_{j\in N, j\neq i} d_{ij}}{n-1}$ Where L_i is the average distance between node i and all other nodes	Average of the path lengths of all nodes. Path lengths are defined as the length of the shortest path between those nodes.
Small World Index	$S = \frac{C/C_{random}}{L/L_{random}}$ Where C and C_{random} are the clustering coefficients, and L and L_{random} are the characteristic path lengths of the respective tested network and a random network. Small-world networks often have $S \gg 1$	A small-world graph has a similar characteristic path length as a random graph with the same degree distribution but is significantly more clustered.
Modularity	$Q = \sum_{u\in M}\left[e_{uu} - \left(\sum_{v\in M} e_{uv}\right)^2\right]$ where the network is fully subdivided into a set of nonoverlapping modules M, and e_{uv} is the proportion of all links that connect nodes in module u with nodes in module v	Modularity is a measure of the structure of a graph, measuring the density of connections within a module or community. Graphs with a high modularity score will have many connections within a community but only few pointing outwards to other communities.
Local Efficiency	$E_{loc} = \frac{1}{n}\sum_{i\in N} E_{loc,i}$	Average of the local efficiency of all nodes

Supplementary Table 3: Nodal graph metrics definition

Metric	*Formula*	*Description*
Degree Centrality	$k_i = \sum_{j \in N} a_{ij}$	Total number of edges connected to a node.
Betweenness Centrality	$b_i = \frac{1}{(n-1)(n-2)} \sum_{\substack{h,j \in N \\ h \neq j, h \neq i, j \neq i}} \frac{p_{hj}^{(i)}}{p_{hj}}$ Where ρ_{hj} is the number of shortest paths between *h* and *j*, and $\rho_{hj}(i)$ is the number of shortest paths between *h* and *j* that pass through *i*	Fraction of all shortest paths in the graph that pass through a node. Nodes with high values of betweenness centrality participate in a large number of shortest paths.
Clustering Coefficient	$C_i = \frac{1}{n} \sum_{i \in N} \frac{2t_i}{k_i(k_i - 1)}$	The clustering coefficient is calculated as the ratio between the number of triangles present around a node and the maximum number of triangles that could possibly be formed around that node.
Nodal Efficiency	$E_{loc,i} = \frac{\sum_{j,h \in N, j \neq i} a_{ij} a_{ih} \left[d_{jh}(N_i)\right]^{-1}}{k_i(k_i - 1)}$	Nodal efficiency is the average of the inverse shortest path length from a node to all other nodes.

* *N* is the set of all nodes in the network, and *n* is the number of nodes. *L* is the set of all links in the network, and *l* is number of links. (*i,j*) is a link between nodes *i* and *i*, (*i,j* ∈ N). a_{ij} is the connection status between *i* and *j*: a_{ij}= 1 when link (*i,j*) exists (when *i* and *j* are neighbours); a_{ij}= 0 otherwise (a_{ii} = 0 for all *i*). We compute the number of links as $l = \sum_{i,j \in N} a_{ij}$ (to avoid ambiguity with directed links we count each undirected link twice, as a_{ij} and as a_{ji})

Supplementary Table 4: LME Formulas and contrasts used in statistical analysis:

Group	LME Formula	Contrasts
All	y ~ 1 + AGE + (1|SUBJECT)[1]	AGE+, AGE-
Female		
Male		
All	y ~ 1 + AGE * GENDER + (1|SUBJECT)[2]	AGE_x_GENDER+, AGE_x_GENDER-
All	y ~ 1 + AGE * GENDER + (1|SUBJECT)[3]	AGE+, AGE-

y, is the response variable which is r-to-z score transformed Pearson's correlation coefficient for Atlas Based Functional Connectivity Analysis, and nodal/global graph measure value for Graph Network Analysis. *AGE* is animal age in days (Date of the MR scan – Date of birth), and *GENDER* is animal gender (Female and Male).

*1) Effect of AGE in 3 different groups, not regarding GENDER as an effect.

*2) Effect of AGE and GENDER interaction.

*3) Effect of AGE, regarding an interaction between AGE and GENDER.

Supplementary Figure 2:

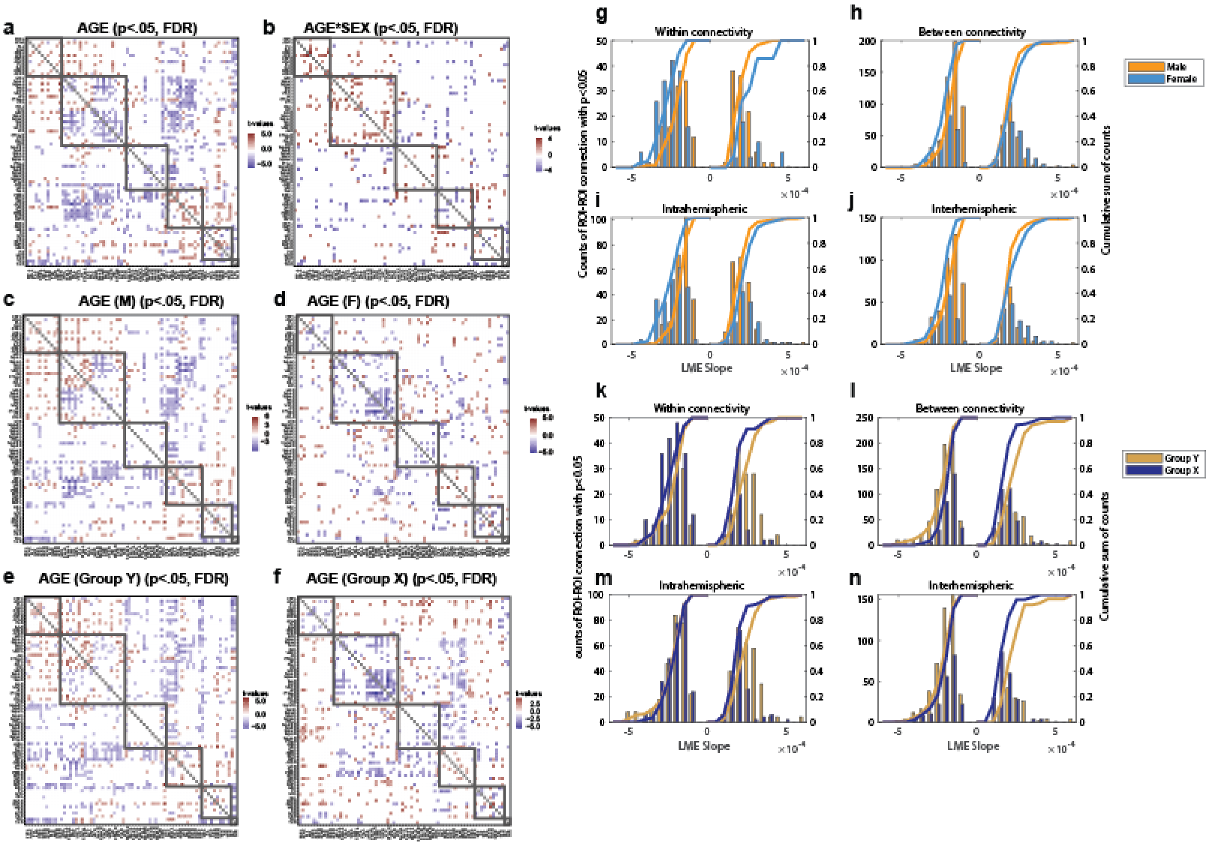

Comparing changes in ROI-ROI correlations with linear mixed effect models shows significant effects of age on the connectivity. In **a,** age was modelled as fixed effect with the individual mouse as random effects (p<0.05, FDR corrected). The Louvain module groups at 12 months are denoted by black squares. A total of 1266 (24.4%) ROI-ROI/ edge correlations showed significant modulation with time and the majority showing negative correlations (62%). Including sex as an additional fixed factor (**b**) showed that many of these significant interactions were also modulated by the factor sex (p<0.05, FDR corrected). Separating males (**c**) from females (**d**) showed that most of these significant changes were due to changes in males (thresholded at p<0.05, FDR corrected). The results were enhanced when we used the classification into group Y (**e**) and X (**f**), with group X showing larger within module decreases and between module increases in functional connectivity. To better understand the nature of the ROI-ROI correlations we looked at the within and between module correlation distributions. In **g-n** we plotted the histograms (left axis) with their cumulative distribution functions (right axis) according to sex and group Y/X.

The strongest effects (ks2stat 0.41, p< 0.0001) on slope decreases were found in within module connectivity in females (**g**), mainly due to a decrease (ks2stat 0.41, p< 0.0001) in intrahemispheric connectivity (**i**). The largest number of changes were observed in males in the between module connectivity (518 vs. 228 counts) and these were mainly due to decreases (**j**) in interhemispheric connections (384 counts in males vs. 152 in females). After regrouping into Y and X we found the strongest effects (ks2stat 0.48, p< 0.0001) on slope increases in within module connectivity in group Y (**k**), mainly due to a increase (ks2stat 0.38, p< 0.0001) in intrahemispheric connectivity (**m**). The largest number of changes were observed in group Y in the between module connectivity (**l**: 660 vs. 300 counts) and these were mainly due to decreases (**n**) in interhemispheric connections (474 counts in Y vs. 204 in X). **g-n** plots the slopes/coefficients of connections with p<0.05 (FDR corrected).

Supplementary Figure 3: Animal weights

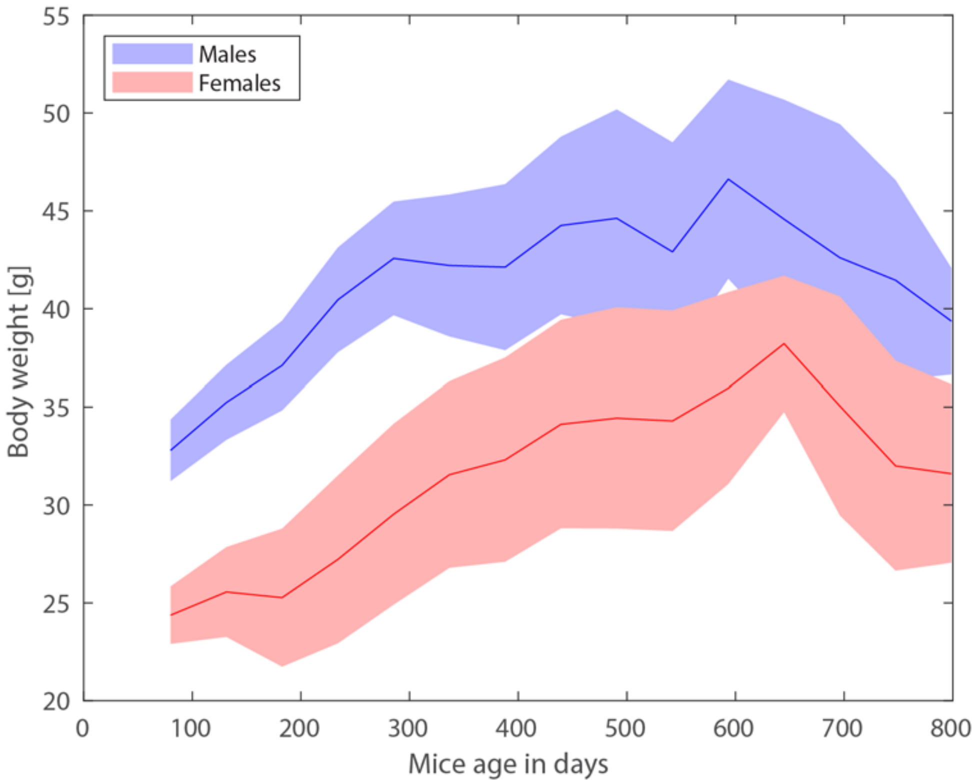

Mice generally gained weight during the study with males achieving larger weights than females (maximal of 56 vs. 44g), which approximately reflected the initial body weight differences at three months (average male 35 vs. female 25g). Males had a faster weight increase, while females maintained a longer constant increase. Males reached a maximal weight at around 19.5 months (593 days) with females showing a later maximum at 21 months (644 days). Thereafter, males showed age related weight loss earlier than females. Confidence intervals are plotted as standard deviations of body weights.
