# Supplementary material for: Longitudinal monitoring of the mouse brain reveals heterogenous network trajectories during aging
Source: Commun Biol. 2024 Feb 20;7:210. doi: 10.1038/s42003-024-05873-8 (PMC10879497; doi:10.1038/s42003-024-05873-8)
Supplement: Supplementary file 2 — Description of Additional Supplementary Files [file 42003_2024_5873_MOESM2_ESM.pdf]

Description of Additional Supplementary Files

File name: Supplementary Data 1
Description: Individuals with descriptive information and scan dates, anesthesia types, number of anesthesia and exclusion criteria.

File name: Supplementary Data 2
Description: Full ROI name, ROI ID (as used in the figures), ROI ID from Grandjean et al., 2020, ROI abbreviation, network module information (ID, % correct labelled).
