# Supplementary material for: Longitudinal monitoring of the mouse brain reveals heterogenous network trajectories during aging
Source: Commun Biol. 2024 Feb 20;7:210. doi: 10.1038/s42003-024-05873-8 (PMC10879497; doi:10.1038/s42003-024-05873-8)
Supplement: Supplementary file 5 — Reporting Summary [file 42003_2024_5873_MOESM5_ESM.pdf]

nature portfolio

Corresponding author(s): Fahad Sultan

Last updated by author(s): 16/1/2024

Reporting Summary

Nature Portfolio wishes to improve the reproducibility of the work that we publish. This form provides structure for consistency and transparency in reporting. For further information on Nature Portfolio policies, see our Editorial Policies and the Editorial Policy Checklist.

Statistics

For all statistical analyses, confirm that the following items are present in the figure legend, table legend, main text, or Methods section.

n/a	Confirmed	
☐	☒	The exact sample size (*n*) for each experimental group/condition, given as a discrete number and unit of measurement
☐	☒	A statement on whether measurements were taken from distinct samples or whether the same sample was measured repeatedly
☐	☒	The statistical test(s) used AND whether they are one- or two-sided<br>*Only common tests should be described solely by name; describe more complex techniques in the Methods section.*
☐	☒	A description of all covariates tested
☐	☒	A description of any assumptions or corrections, such as tests of normality and adjustment for multiple comparisons
☐	☒	A full description of the statistical parameters including central tendency (e.g. means) or other basic estimates (e.g. regression coefficient) AND variation (e.g. standard deviation) or associated estimates of uncertainty (e.g. confidence intervals)
☐	☒	For null hypothesis testing, the test statistic (e.g. *F*, *t*, *r*) with confidence intervals, effect sizes, degrees of freedom and *P* value noted<br>*Give P values as exact values whenever suitable.*
☒	☐	For Bayesian analysis, information on the choice of priors and Markov chain Monte Carlo settings
☐	☒	For hierarchical and complex designs, identification of the appropriate level for tests and full reporting of outcomes
☐	☒	Estimates of effect sizes (e.g. Cohen's *d*, Pearson's *r*), indicating how they were calculated

Our web collection on statistics for biologists contains articles on many of the points above.

Software and code

Policy information about availability of computer code

Data collection	Bruker ParaVision v6.0.1
Data analysis	FMRIB Software Library (FSL) v6.0<br>Advanced Normalization Tools (ANTs) v2.3.4.dev192-g5b7c0<br>Skullstripper v2.0<br>AFNI - Analysis of Functional NeuroImages v21.2.04<br>R v4.1.2<br>Matlab R2021b (9.11.0.1769968)<br>GRETNA v2.0<br>Brain Connectivity Toolbox v2019-03-03

For manuscripts utilizing custom algorithms or software that are central to the research but not yet described in published literature, software must be made available to editors and reviewers. We strongly encourage code deposition in a community repository (e.g. GitHub). See the Nature Portfolio guidelines for submitting code & software for further information.

Data

Policy information about availability of data

All manuscripts must include a data availability statement. This statement should provide the following information, where applicable:

- Accession codes, unique identifiers, or web links for publicly available datasets
- A description of any restrictions on data availability
- For clinical datasets or third party data, please ensure that the statement adheres to our policy

Exemplary functional and structural MRI raw data are available via the OSF (Sultan, F. (2024, January 16). Longitudinal monitoring of aging mouse brain. Retrieved from osf.io/h4dkp) data sharing service or upon reasonable request. The scripts employed for data analysis will be uploaded to Github.

Research involving human participants, their data, or biological material

Policy information about studies with human participants or human data. See also policy information about sex, gender (identity/presentation), and sexual orientation and race, ethnicity and racism.

Reporting on sex and gender

Use the terms sex (biological attribute) and gender (shaped by social and cultural circumstances) carefully in order to avoid confusing both terms. Indicate if findings apply to only one sex or gender; describe whether sex and gender were considered in study design; whether sex and/or gender was determined based on self-reporting or assigned and methods used.
Provide in the source data disaggregated sex and gender data, where this information has been collected, and if consent has been obtained for sharing of individual-level data; provide overall numbers in this Reporting Summary. Please state if this information has not been collected.
Report sex- and gender-based analyses where performed, justify reasons for lack of sex- and gender-based analysis.

Reporting on race, ethnicity, or other socially relevant groupings

Please specify the socially constructed or socially relevant categorization variable(s) used in your manuscript and explain why they were used. Please note that such variables should not be used as proxies for other socially constructed/relevant variables (for example, race or ethnicity should not be used as a proxy for socioeconomic status).
Provide clear definitions of the relevant terms used, how they were provided (by the participants/respondents, the researchers, or third parties), and the method(s) used to classify people into the different categories (e.g. self-report, census or administrative data, social media data, etc.)
Please provide details about how you controlled for confounding variables in your analyses.

Population characteristics

Describe the covariate-relevant population characteristics of the human research participants (e.g. age, genotypic information, past and current diagnosis and treatment categories). If you filled out the behavioural & social sciences study design questions and have nothing to add here, write "See above."

Recruitment

Describe how participants were recruited. Outline any potential self-selection bias or other biases that may be present and how these are likely to impact results.

Ethics oversight

Identify the organization(s) that approved the study protocol.

Note that full information on the approval of the study protocol must also be provided in the manuscript.

Field-specific reporting

Please select the one below that is the best fit for your research. If you are not sure, read the appropriate sections before making your selection.

☒ Life sciences ☐ Behavioural & social sciences ☐ Ecological, evolutionary & environmental sciences

For a reference copy of the document with all sections, see nature.com/documents/nr-reporting-summary-flat.pdf

Life sciences study design

All studies must disclose on these points even when the disclosure is negative.

Sample size

This study had a planned longitudinal design with four animals added as cross-sectional to increase the very old female population (24 months). The sample size was initially calculated as n=22 to detect a 5% alpha error with effect size of 0.4 based on previous fMRI aging change estimates in mice (A Egimendia et al., Frontiers in Aging Neuroscience 11 (2019). However, previous studies have only relied on short follow-up time points so to derive a precisely estimated effect size is difficult. To ensure that we had a sufficient sample size throughout the lifespan, we supplemented our sample with an additional 43 mice due to the loss of some animals and had 36 at the final stage (24 months).

Data exclusions

Six MRI scans were excluded in full due to the presence of artifacts or acquisition errors. These are listed in Supplementary table 1 and 2. Fourteen scans were obtained with one scan only (instead of two scans as in the remaining 128) due the presence of artifacts or acquisition errors in the other.
Six mice revealed a very low segregation index at 12 and 18 months (below 0.43). These mice furthermore either developed neurological symptoms or died early on of unknown reasons (listed in Supplementary table 1 and 2). These mice were therefore also excluded from further analysis.

Replication	Replication was performed by grouping mice into smaller subgroups (grouping based on cage affiliation and group size).
Randomization	Not applied
Blinding	Not applied

Behavioural & social sciences study design

All studies must disclose on these points even when the disclosure is negative.

Study description	*Briefly describe the study type including whether data are quantitative, qualitative, or mixed-methods (e.g. qualitative cross-sectional, quantitative experimental, mixed-methods case study).*
Research sample	*State the research sample (e.g. Harvard university undergraduates, villagers in rural India) and provide relevant demographic information (e.g. age, sex) and indicate whether the sample is representative. Provide a rationale for the study sample chosen. For studies involving existing datasets, please describe the dataset and source.*
Sampling strategy	*Describe the sampling procedure (e.g. random, snowball, stratified, convenience). Describe the statistical methods that were used to predetermine sample size OR if no sample-size calculation was performed, describe how sample sizes were chosen and provide a rationale for why these sample sizes are sufficient. For qualitative data, please indicate whether data saturation was considered, and what criteria were used to decide that no further sampling was needed.*
Data collection	*Provide details about the data collection procedure, including the instruments or devices used to record the data (e.g. pen and paper, computer, eye tracker, video or audio equipment) whether anyone was present besides the participant(s) and the researcher, and whether the researcher was blind to experimental condition and/or the study hypothesis during data collection.*
Timing	*Indicate the start and stop dates of data collection. If there is a gap between collection periods, state the dates for each sample cohort.*
Data exclusions	*If no data were excluded from the analyses, state so OR if data were excluded, provide the exact number of exclusions and the rationale behind them, indicating whether exclusion criteria were pre-established.*
Non-participation	*State how many participants dropped out/declined participation and the reason(s) given OR provide response rate OR state that no participants dropped out/declined participation.*
Randomization	*If participants were not allocated into experimental groups, state so OR describe how participants were allocated to groups, and if allocation was not random, describe how covariates were controlled.*

Ecological, evolutionary & environmental sciences study design

All studies must disclose on these points even when the disclosure is negative.

Study description	*Briefly describe the study. For quantitative data include treatment factors and interactions, design structure (e.g. factorial, nested, hierarchical), nature and number of experimental units and replicates.*
Research sample	*Describe the research sample (e.g. a group of tagged Passer domesticus, all Stenocereus thurberi within Organ Pipe Cactus National Monument), and provide a rationale for the sample choice. When relevant, describe the organism taxa, source, sex, age range and any manipulations. State what population the sample is meant to represent when applicable. For studies involving existing datasets, describe the data and its source.*
Sampling strategy	*Note the sampling procedure. Describe the statistical methods that were used to predetermine sample size OR if no sample-size calculation was performed, describe how sample sizes were chosen and provide a rationale for why these sample sizes are sufficient.*
Data collection	*Describe the data collection procedure, including who recorded the data and how.*
Timing and spatial scale	*Indicate the start and stop dates of data collection, noting the frequency and periodicity of sampling and providing a rationale for these choices. If there is a gap between collection periods, state the dates for each sample cohort. Specify the spatial scale from which the data are taken*
Data exclusions	*If no data were excluded from the analyses, state so OR if data were excluded, describe the exclusions and the rationale behind them, indicating whether exclusion criteria were pre-established.*
Reproducibility	*Describe the measures taken to verify the reproducibility of experimental findings. For each experiment, note whether any attempts to repeat the experiment failed OR state that all attempts to repeat the experiment were successful.*
Randomization	*Describe how samples/organisms/participants were allocated into groups. If allocation was not random, describe how covariates were controlled. If this is not relevant to your study, explain why.*

Blinding | Describe the extent of blinding used during data acquisition and analysis. If blinding was not possible, describe why OR explain why blinding was not relevant to your study.

Did the study involve field work? ☐ Yes ☒ No

Field work, collection and transport

Field conditions	*Describe the study conditions for field work, providing relevant parameters (e.g. temperature, rainfall).*
Location	*State the location of the sampling or experiment, providing relevant parameters (e.g. latitude and longitude, elevation, water depth).*
Access & import/export	*Describe the efforts you have made to access habitats and to collect and import/export your samples in a responsible manner and in compliance with local, national and international laws, noting any permits that were obtained (give the name of the issuing authority, the date of issue, and any identifying information).*
Disturbance	*Describe any disturbance caused by the study and how it was minimized.*

Reporting for specific materials, systems and methods

We require information from authors about some types of materials, experimental systems and methods used in many studies. Here, indicate whether each material, system or method listed is relevant to your study. If you are not sure if a list item applies to your research, read the appropriate section before selecting a response.

Materials & experimental systems

n/a	Involved in the study	
☒	☐	Antibodies
☒	☐	Eukaryotic cell lines
☒	☐	Palaeontology and archaeology
☐	☒	Animals and other organisms
☒	☐	Clinical data
☒	☐	Dual use research of concern
☒	☐	Plants

Methods

n/a	Involved in the study	
☒	☐	ChIP-seq
☒	☐	Flow cytometry
☐	☒	MRI-based neuroimaging

Animals and other research organisms

Policy information about studies involving animals; ARRIVE guidelines recommended for reporting animal research, and Sex and Gender in Research

Laboratory animals	C57Bl/6J mice
Wild animals	*Provide details on animals observed in or captured in the field; report species and age where possible. Describe how animals were caught and transported and what happened to captive animals after the study (if killed, explain why and describe method; if released, say where and when) OR state that the study did not involve wild animals.*
Reporting on sex	*Indicate if findings apply to only one sex; describe whether sex was considered in study design, methods used for assigning sex. Provide data disaggregated for sex where this information has been collected in the source data as appropriate; provide overall numbers in this Reporting Summary. Please state if this information has not been collected. Report sex-based analyses where performed, justify reasons for lack of sex-based analysis.*
Field-collected samples	*For laboratory work with field-collected samples, describe all relevant parameters such as housing, maintenance, temperature, photoperiod and end-of-experiment protocol OR state that the study did not involve samples collected from the field.*
Ethics oversight	All procedures performed in this study were approved by the regional Animal Research Ethics Committee of Northern Norrland and by the Swedish Board of Agriculture (Ethical permit number: A17-2019).

Note that full information on the approval of the study protocol must also be provided in the manuscript.

Magnetic resonance imaging

Experimental design

Design type	Resting-state fMRI

Design specifications	420 volumes were acquired for resting-state fMRI
Behavioral performance measures	No behavioral perfromances were measured.

Acquisition

Imaging type(s)	functional MRI
Field strength	9.4T
Sequence & imaging parameters	BOLD activity from these slices was acquired at a temporal resolution of 1.5 seconds per volume with single-shot GE-recalled EPI images (TR/TE=1500/22ms, bandwidth=208.33kHz, FA=60 , FOV=16.2mm×9.6mm, Matrix=54x32) and a total of 420 volumes.
Area of acquisition	Whole brain

Diffusion MRI ☐ Used ☒ Not used

Preprocessing

Preprocessing software	FMRIB Software Library (FSL) v6.0<br>Advanced Normalization Tools (ANTs) v2.3.4.dev192-g5b7c0<br>Skullstripper v2.0<br>AFNI - Analysis of Functional NeuroImages v21.2.04
Normalization	The functional data was linearly registered to the Allen Reference Atlas (Mouse Brain Common Coordinate Framework v3.0 available from atlas.brain-map.org) (Wang et al 2020a) and was performed with FLIRT (FSL).
Normalization template	Allen Mouse Brain Atlas
Noise and artifact removal	Six estimated motion correction parameters, white matter (WM), ventricle (VEN) signals as well as mean global signal and its linear and quadratic derivatives were regressed from time series (3dDetrend, AFNI) to reduce the effects of physiological noise and motion. A high-pass temporal filter (>0.01Hz) was applied to the time series rather than a band-pass filter, since it is shown that valuable signal might be present in higher frequencies
Volume censoring	First five volumes were omitted.

Statistical modeling & inference

Model type and settings	ROI-to-ROI correlations at the three time points were analyzed using linear mixed effects (LME) model using lme4 v1.1-27.1 package (Bates et al 2015, Bernal-Rusiel et al 2013) for R v4.1.2 (R Core Team 2014).
Effect(s) tested	Sex and age were studied as fixed effects, and subject as random effect using maximum likelihood estimations.

Specify type of analysis: ☐ Whole brain ☐ ROI-based ☒ Both

Anatomical location(s)	72 ROIs from Allen Mouse Brain Atlas were selected based on Grandjean et al. (Grandjean et al 2020). This atlas is described in the study by Lein and colleagues (Lein et al 2007) and is based on a modified version of the Swanson (Swanson 2004) and Hof atlases (Hof 2000). The atlas rois and the description files are available under Github repository, https://github.com/grandjeanlab/mergeallen.
Statistic type for inference<br>(See Eklund et al. 2016)	Atlas ROI based whole brain analysis.
Correction	FDR corrections were applied.

Models & analysis

n/a	Involved in the study
☐	☒ Functional and/or effective connectivity
☐	☒ Graph analysis
☐	☒ Multivariate modeling or predictive analysis

Functional and/or effective connectivity	Allen atlas ROI based functional connectivity with linear-mixed effect models of chaneg over time.
Graph analysis	Louvain modules, Global and nodal graph measures

Multivariate modeling and predictive analysis	Linear mixed effects modeling of time and sex
